# Construction and Immunogenicity of a Recombinant Pseudorabies Virus Expressing SARS-CoV-2-S and SARS-CoV-2-N

**DOI:** 10.3389/fvets.2022.920087

**Published:** 2022-08-02

**Authors:** Ruoying Li, Guanming Shao, Zi Xie, Zezhong Hu, Keyu Feng, Jiahui He, Hailong Wang, Jun Fu, Xinheng Zhang, Qingmei Xie

**Affiliations:** ^1^Heyuan Branch, Guangdong Provincial Laboratory of Lingnan Modern Agricultural Science and Technology & Guangdong Provincial Key Lab of Agro-Animal Genomics and Molecular Breeding, College of Animal Science, South China Agricultural University, Guangzhou, China; ^2^Guangdong Engineering Research Center for Vector Vaccine of Animal Virus, College of Animal Science, South China Agricultural University, Guangzhou, China; ^3^South China Collaborative Innovation Center for Poultry Disease Control and Product Safety, College of Animal Science, South China Agricultural University, Guangzhou, China; ^4^State Key Laboratory of Microbial Technology, Institute of Microbial Technology, Helmholtz International Lab for Anti-infectives, Shandong University–Helmholtz Institute of Biotechnology, Shandong University, Jinan, China; ^5^Key Laboratory of Animal Health Aquaculture and Environmental Control, College of Animal Science, South China Agricultural University, Guangzhou, China

**Keywords:** pseudorabies virus, SARS-CoV-2-N, SARS-CoV-2-S, Red/ET recombination technique, recombinant virus

## Abstract

Coronavirus (CoV) is an important pathogen of humans and animals, which can infect humans or animals through the respiratory mucosal route. Syndrome coronavirus 2 (SARS-CoV-2) is quite similar to syndrome coronavirus (SARS-CoV) with the same receptor, angiotensin-converting enzyme 2 (ACE2). The S and N proteins are the most important protective antigens of the SARS-CoV-2. The S protein on the viral membrane mediates the virus attachment with the host cells, and the N protein is the most abundant expression during infection. In this study, the recombinant viruses expressing the S and N proteins of SARS-CoV-2 were successfully constructed by Red/ET recombinant technology using Pseudorabies virus (PRV) strain Bartha-K61 as a vector. Genetic stability and growth kinetics analysis showed that the recombinant viruses rPRV-SARS-CoV-2-S and rPRV-SARS-CoV-2-N had similar genetic stability and proliferation characteristics to the parental PRV. The immunoassay results showed that mice immunized with recombinant viruses could produce total IgG antibodies. Therefore, PRV is feasible and promising as a viral vector to express SARS-CoV-2-S and SARS-CoV-2-N genes. This study can provide a reference for future research on live vector vaccines for domestic animals, pets, and wild animals.

## Introduction

The severe acute respiratory syndrome coronavirus 2 (SARS-CoV-2), first reported by China in December 2019, has spread throughout the world. On January 30, 2020, World Health Organization (WHO) officially declared SARS-CoV-2 as a public health emergency of international concern. SARS-CoV-2 mainly spreads among humans but can also spread among animals and from humans to animals. A cat in Belgian that had contact with a human SARS-CoV-2 patient tested positive for SARS-CoV-2 (1). Dogs in Hong Kong were infected in a human household with SARS-CoV-2 (2). Data suggests that cats and dogs are naturally susceptible to SARS-CoV-2 infection, and cats are more susceptible to SARS-CoV-2 infection and may transmit the virus to other cats (3). Therefore, monitoring SARS-CoV-2 infection in animal species and vaccine preparation is critical.

Coronaviruses (CoVs)-enveloped positive-sense RNA viruses, belong to the Coronaviridae family of the Orthocoronavirinae subfamily, can infect birds, mammals, and humans (4). The viral genome is about 27–32 kb, which encodes for non-structural proteins and four major structural proteins: spike (S), membrane (M), envelope (E), and nucleocapsid (N) proteins (5). The genome phylogenetic analysis suggests that SARS-CoV-2 is quite similar to SARS-CoV, and both of them use the same receptor, angiotensin-converting enzyme 2 (ACE2), to infect humans, but SARS-CoV-2 has a stronger ability to transmit from human to human (6, 7).

Coronavirus infection is based on the interaction between the receptor-binding domain (RBD) located in the S1 subunit of spike protein and the target receptor on the host cell surface (8). The S protein presents on the viral membrane and mediates the virus attachment with the host cells (9).Hence, the S protein is considered a key target for SARS-CoV-2 vaccine development, as it can induce neutralizing antibodies that prevent viral uptake through the human ACE2 receptor (10, 11). Previous research on SARS-CoV had shown that the protection from infection in mouse models was attributed to antibody responses generated against the S protein (12–14). In addition, multiple studies suggested that the N protein of SARS-CoV, abundantly expressed protein during infection, appears to be highly immunogenic (15). Relevant data have shown that antibodies generated against the N protein of SARS-CoV in over 94% of samples from SARS study patients (16).

Pseudorabies virus (PRV) belongs to the subfamily Alphaherpesvirinae of the Herpesviridae family (17). The genome of PRV, a double-stranded linear DNA molecule about 143 kb in size, contains almost 70 open reading frames (ORFs) encoding for more than 70 viral proteins (18). Multiple studies have shown that several genes are not essential for viral replication, including glycoprotein E (gE), glycoprotein I (gI), glycoprotein G (gG), thymidine kinase (TK), and protein kinase (PK), which are the possible insertion sites for heterologous genes without affecting virus immunogenicity or propagation (19–21). Bartha-K61 strain, whose safety has been widely recognized, is a classic PRV vaccine strain (22). It has a long clinical application history, a wide range of applications, and a large quantity. The host range of PRV is very wide, for PRV infects pigs, dogs, sheep, cattle, cats, rabbits, minks, and other animals (23, 24). Due to its safety and host diversity, PRV Bartha-K61 was selected as the vaccine vector in this study.

The above researches render PRV Bartha-K61 a promising live vector for the development of multivalent vaccines to provide protection against PR and other diseases. Therefore, in the present study, we constructed a recombinant pseudorabies virus expressing the S protein and the N protein of SARS-CoV-2 and evaluated the growth properties, genetic stability, and immunogenicity of the recombinant viruses. This study will provide a reference for the development of SARS-CoV-2 vector vaccines.

## Materials and Methods

### Animal Rights Statement

The animal experiments were carried out in strict accordance with the recommendations in the Guide for the Care and Use of Laboratory Animals of the Ministry of Science and Technology of the People's Republic of China. The protocol was approved by the Committee for Animal Experiments (approval ID: SYXK2019-0136) of the South China Agricultural University.

### Experimental Animal

The experiment on thirty-two 3-month-old female BALB/c mice was provided by the Laboratory Animal Center of Southern Medical University.

### Viruses and Cells

PRV strain Bartha-K61 was preserved by our lab; Vero and porcine kidney (PK)-15 cells were purchased from the American Type Culture Collection (ATCC, Rockville, MD) and cultured in the Dulbecco's modified Eagle's medium (DMEM) supplemented with 10% heat-inactivated fetal bovine serum (FBS), plus 1% Antibiotic-Antimycotic (10,000 I.U./mL of penicillin, 10,000 μg/mL of streptomycin) and incubated at 37°C with 5% CO_2_.

### Construction of the Recombinant Plasmids

The pUC57-SARS-CoV-2-S and pUC57-SARS-CoV-2-N were synthesized by Sangon Biotech (Shanghai, China) according to the nucleotide sequence of the Wuhan-Hu-1 strain (Accession number: MN908947) published by Genebank. The vector pBeloBAC11-cm was provided by Helmholtz International Lab for Anti-infectives, Shandong University. The plasmid pBeloBAC-PRV-Bartha-K61 was provided by our lab. The vector was homologously recombined with the whole genome of PRV by RecET homologous recombination technique. Then, the infectious clone plasmid was obtained and was named pBeloBAC-PRV-Bartha-K61.

**Figure 1:**
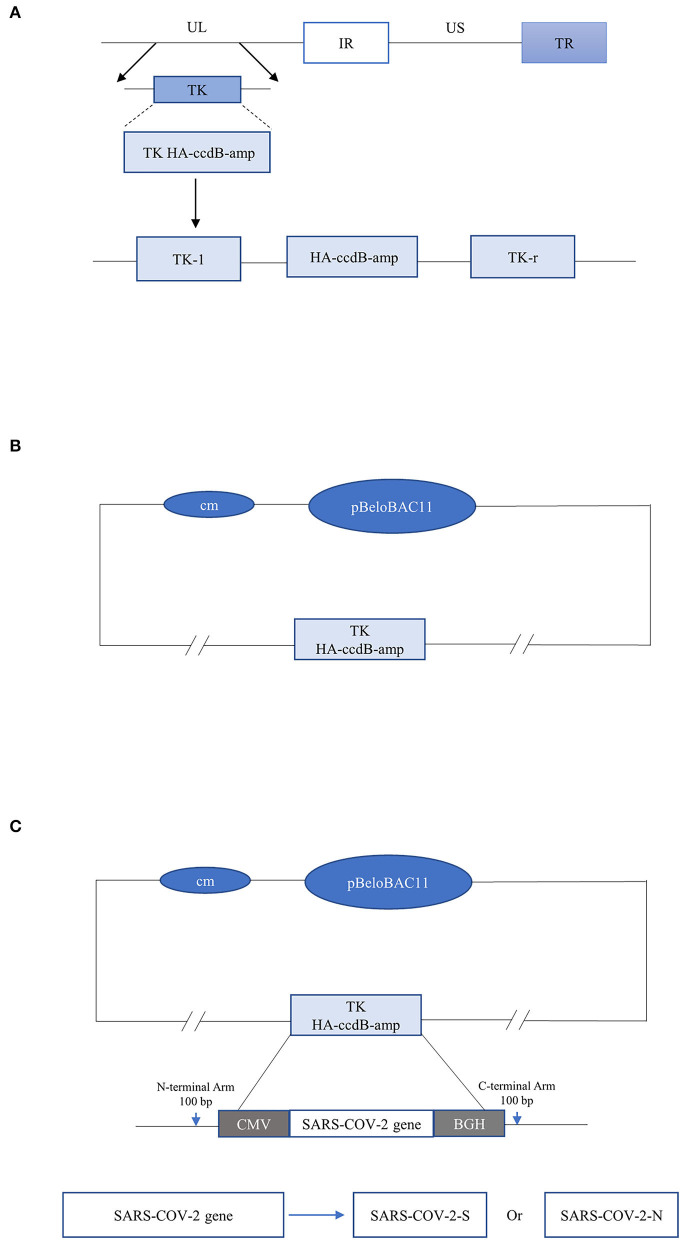
**(A)** The selectable gene TK HA-ccdB-amp containing the homologous arm of TK gene of PRV vaccine Strain Bartha-K61 was amplified by PCR. **(B)** In the first step, the selectable gene TK HA-ccdB-amp, and plasmid pBeloBAC-PRV-Bartha-K61 were recombinant by the Red/ET recombination technique. The TK site of the Bartha-K61 strain was replaced with the selectable gene. **(C)** In the second step, the gene SARS-CoV-2-S and SARS-CoV-2-N were recombined with pBeloBAC-PRV-Bartha-K61, respectively, and then inserted at the TK site, driven by the CMV promoter and ended by BGH termination signal. Finally, the TK site was replaced with SARS-CoV-2-S and SARS-CoV-2-N.

Recombinant pseudorabies viruses were constructed by a two-step modification method. In the first step, the TK HA-ccdB-amp gene containing the homologous arm of the TK site of PRV strain Bartha-K61 was amplified by PCR. The selectable gene TK HA-ccdB-amp was recombined with the plasmid pBeloBAC- PRV-Bartha-K61 by the Red/ET recombination technique. The TK site was replaced by the selectable gene. The recombinant plasmid was obtained by forwarding selection and was named pBeloBAC-cm-PRV-Bartha-ΔTK/ccdB-amp. In the second step, SARS-CoV-2 antigen S and N genes were amplified by PCR, respectively. The sequence was added to the CMV promoter at the 5' end, BGH terminator at the 3' end, and homologous arms of the TK site at both ends by fusion PCR. The antigen gene S and N were recombined with the above recombinant plasmid, respectively, by the Red/ET recombination technique. Recombinant plasmids were obtained by reverse selection and were named pBeloBAC11-PRV-SARS-CoV-2-S and pBeloBAC11-PRV-SARS-CoV-2-N (Figure 1).

### Rescue of the Recombinant Virus

The parental virus rPRV-Bartha-K61 has been rescued. In this study, the plasmid pBeloBAC11-PRV-SARS-CoV-2-S and pBeloBAC11-PRV-SARS-CoV-2-N were transfected into the Vero cells according to the instructions for Lipofectamine 3000 Reagent (Invitrogen), respectively. The supernatants were collected with 80% of cytopathogenic changes in the cells and had four rounds of blind passage in Vero cells. The purified recombinant viruses were obtained and named rPRV-SARS-CoV-2-S and rPRV-SARS-CoV-2-N, respectively.

### Western Blot

PK-15 cells were infected with rPRV-SARS-CoV-2-S and rPRV-SARS-CoV-2-N at a multiplicity of infection (MOI) of 1. Cell proteins were collected 48 h post-infection. Cell lysates were subjected to SDS-polyacrylamide gel electrophoresis (SDS-PAGE) and transferred to PVDF membranes. The membranes were blocked in 5% non-fat milk-TBST solution (Amresco, USA) overnight and incubated with the HA tag polyclonal antibody (1:1,000 dilution, produced by Sangong Biotech), followed by incubation with 1:10,000 dilution of the secondary antibody HRP-conjugated anti-mice IgG (Proteintech Group, Inc., USA). The Super ECL Detection Reagent (Epizyme, Shanghai) was added for color development, and the Azure c300 digital imager system (Azure Biosystems, Dublin, CA) was used to image protein bands.

### Genetic Stability

Recombinant viruses rPRV-SARS-CoV-2-S and rPRV-SARS-CoV-2-N were passaged for 10 generations, and the genetic stability of SARS-CoV-2-S and SARS-CoV-2-N were detected in every five generations.

### Growth Kinetics

PK-15 cells were infected with the parental virus rPRV, recombinant viruses rPRV-SARS-CoV-2-S and rPRV-SARS-CoV-2-N, respectively. The supernatants were collected at 12, 24, 36, 48, 60, and 72 h after infection. The 50% tissue culture infective dose (TCID_50_) at each time point was calculated by the Reed-Muench method.

### Protein Expression and Purification

The S protein and the N protein were purchased from Sinobiological, Beijing. The N protein was purified in this study. The recombinant plasmid pET32a-SARS-CoV-2-N was transformed into *E. coli*, and used IPTG to induce protein expression. The protein was purified by Ni-NTA chromatography after lysis and analyzed by SDS-PAGE and Western Blot. The S protein and the purified N protein were then used as an antigen to develop an indirect ELISA (I-ELISA) assay.

### Immunization of Mice With SARS-CoV-2-S and SARS-CoV-2-N

Thirty-two 3-month-old female BALB/c mice were housed in barrier animal facilities at the Laboratory Animal Center of South China Agricultural University. Mice were divided into groups A, B, C, and D, with eight mice in each group. Mice in groups A, B, and C were injected with rPRV-SARS-CoV-2-S, rPRV-SARS-CoV-2-N, and rPRV-Bartha-K61 (1,000 TCID_50_/0.1 mL) by intramuscular injection, respectively. Mice in group D were injected with the same dose of PBS by intramuscular injection. Two weeks later, the mice were strengthened by the second immunization with the same dose as the first immunization.

### The Establishment of Indirect ELISA Based on SARS-CoV-2 S and N Protein

The working concentrations of recombinant antigen (S protein and purified N protein) and their antibodies (Sinobiological, Beijing) for I-ELISA were determined by chequerboard titration (CBT). SARS-CoV-2-S and SARS-CoV-2-N positive and negative serum samples were prepared by our lab. The SARS-CoV-2-S serum samples were diluted 2-folds from 1:100 to 1: 8,000, whereas antigen was diluted 2-folds from 2 μg/mL to 0.0625 μg/mL per well in coating buffer (carbonate/bicarbonate buffer, pH 9.6). The SARS-CoV-2-N serum samples were diluted 2-folds from 1:1,000 to 1: 8,000, whereas antigen was diluted two 2-folds from 8 μg/mL to 0.0625 μg/mL per well in coating buffer. All were used in CBT analysis to determine the optimal dilution of antigen/antibody with the maximum positive and negative values (P/N) in optical absorbance at 450 nm.

In brief, 96-well microtiter ELISA plates (Nunc, USA) were coated with the purified N and S protein diluted in coating buffer (pH 9.6), where 100 μl of coating buffer was added. The plates were then incubated at 4°C overnight and washed three times with PBST washing buffer (PBS containing 0.05 % Tween-20). Then, the plates were blocked by adding 300 μL of blocking buffer (5% skim milk diluted in PBST) to each well and incubated at 37°C for 2 h. After washing, the test serum samples were diluted in PBST and added to 100 μl/well in triplicate. Positive and negative serum samples were included in each run. The plates were incubated at 37°C for 1 h and washed three times, and anti-mice IgG HRPO conjugate (1:2,000 dilution in PBST, 100 μL/well) was added to all the wells of the plate and incubated at 37°C for 1 h. Soluble TMB Substrate Solution (TianGen Biotech, Beijing, China) was added to the plate (100 μL/well) after washing three times and incubated at room temperature for 30 min. The reaction was stopped by adding 2M H_2_SO_4_, 50 μL/well. Then, the optical density value (OD) was measured at 450 nm using a microplate reader (ELx808, BioTek, USA).

### Antibody Detection of SARS-CoV-2-S and SARS-CoV-2-N

After immunization, blood was collected from mice of each group every 7 days. Serum samples (*n* = 192) were isolated from blood and were used to detect antibodies using I-ELISA based on SARS-CoV-2 S and N protein.

### Statistical Analyses

Statistical analysis of the data was performed by the GraphPad Prism software version 8.3.0 (GraphPad Software Inc., San Diego, CA, USA). An unpaired *t*-test was used to evaluate the differences between groups. Data were presented as the mean ± standard deviation. *P* < 0.05 was taken to indicate a statistically significant difference.

## Results

### Identification of Recombinant Plasmids

**Figure 2:**
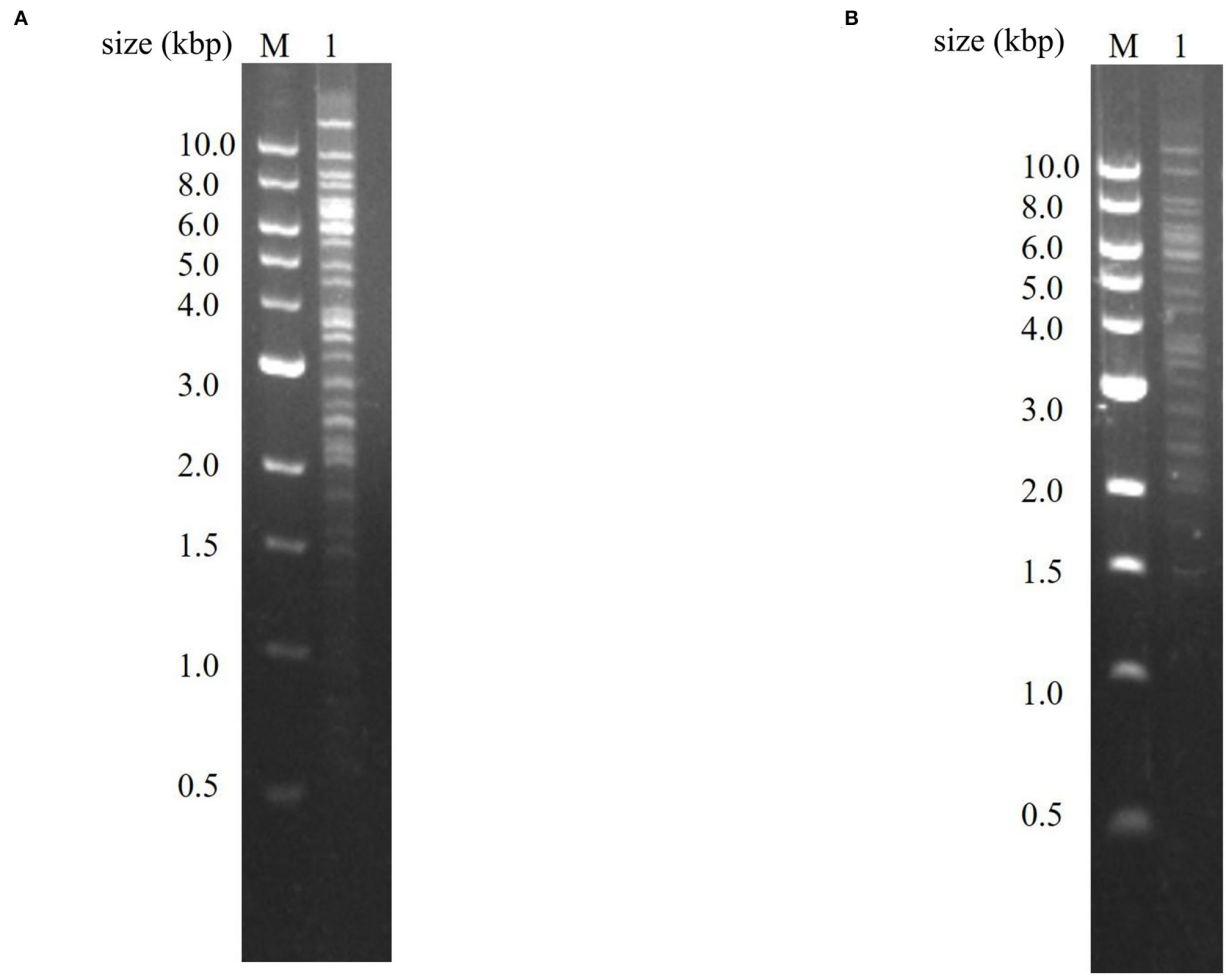
Identification of recombinant plasmids pBeloBAC11-PRV-SARS-CoV-2-S and plasmid pBeloBAC11-PRV-SARS-CoV-N. **(A)** Identification of plasmid pBeloBAC11-PRV-SARS-CoV-2-S. Lane 1 was the PCR product of recombinant plasmid pBeloBAC11-PRV-SARS-CoV-2-S. **(B)** Identification of plasmid pBeloBAC11-PRV-SARS-CoV-2-N. Lane 1 was the PCR product of recombinant plasmid pBeloBAC11-PRV-SARS-CoV-2-N. M was DL10000 DNA Marker (Takara, China).

As shown in Figures 2A,B, the presence of the recombinant plasmids pBeloBAC11-PRV-SARS-CoV-2-S and pBeloBAC11-PRV-SARS-CoV-2-N was detected. The recombinant plasmids were successfully constructed by the Red/ET recombination technology.

### Identification of Recombinant Viruses

**Figure 3:**
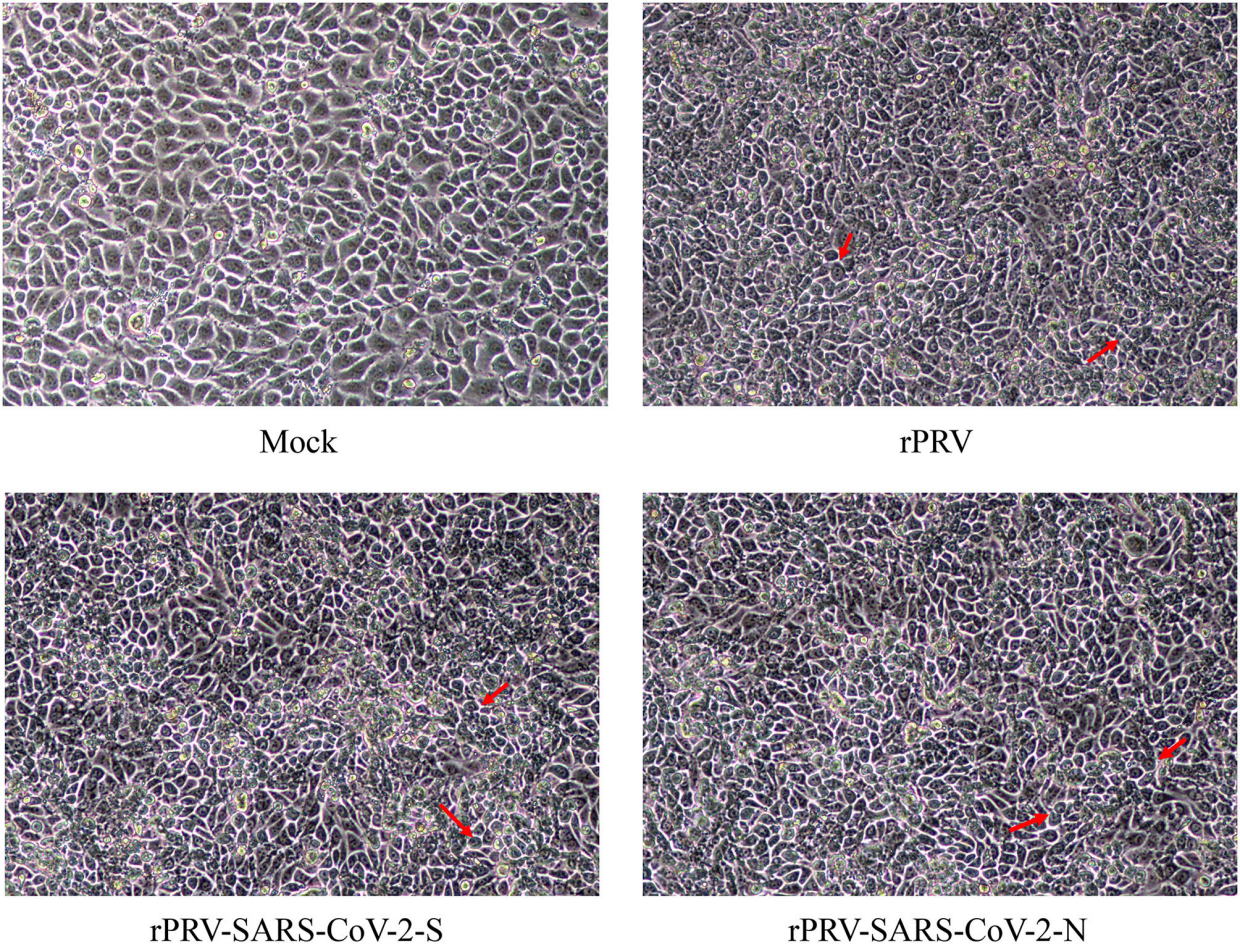
Cytopathic of recombinant viruses in PK-15 cells. PK-15 cells were infected with each recombinant virus at MOI of 1. After 48 h, cytopathic effects were observed. The cells indicated by the red arrow had CPE, which were rounder than the control group.

**Figure 4:**
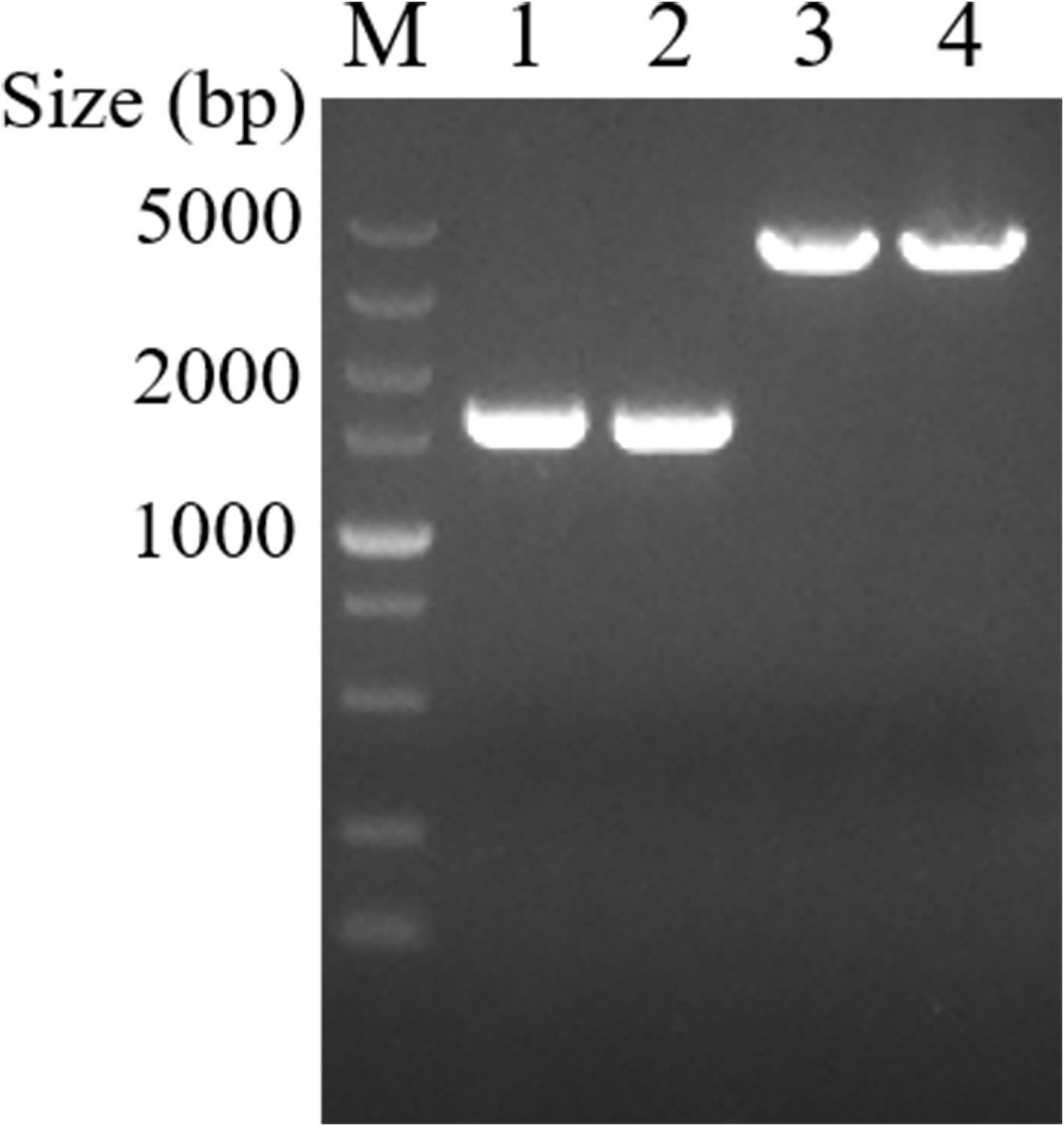
Rescue of recombinant viruses rPRV-SARS-CoV-2-S and rPRV-SARS-CoV-2-N identified by PCR. Lanes 1 and 2 were recombinant viruses rPRV-SARS-CoV-2-N F5 and F10. Lanes 3 and 4 were recombinant viruses rPRV-SARS-CoV-2-S F5 and F10. M was DL5000 DNA Marker (Takara, China).

**Figure 5:**
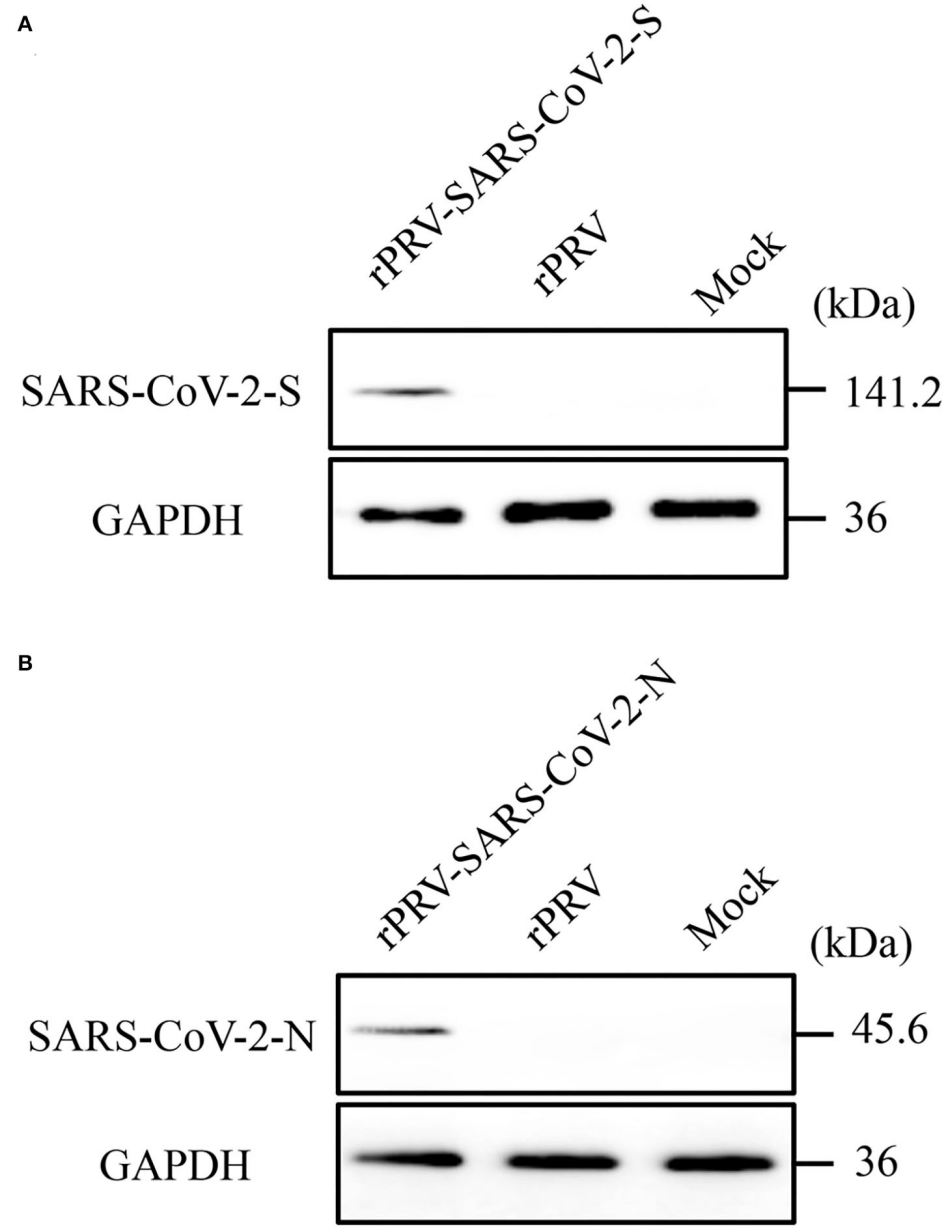
Identification of recombinant viruses rPRV-SARS-CoV-2-S and rPRV-SARS-CoV-2-N by Western blot. After 48h, cell lysates were collected and analyzed by Western Blotting. Recombinant proteins were detected using a HA tag polyclonal antibody (1:1000 dilution) as the primary antibody and a HRP-conjugated anti-mice IgG (1:10000 dilution) as the secondary antibody. GAPDH was used as sample-loading control. **(A)** Protein expression using recombinant PRV-SARS-CoV-2-S. The molecular weight of rPRV-SARS-CoV-2-S was about 141.2 kDa; **(B)** Protein expression using recombinant rPRV-SARS-CoV-2-N. The molecular weight of rPRV-SARS-CoV-2-S was about 45.6 kDa.

The PK-15 cells were infected with the purified recombinant viruses rPRV-SARS-CoV-2-S and rPRV-SARS-CoV-2-N. As shown in Figure 3, PK-15 cells infected with rPRV-Bartha-K61, rPRV-SARS-CoV-2-S and rPRV-SARS-CoV-2-N became rounder than the control. The recombinant viruses rPRV-SARS-CoV-2-S and rPRV-SARS-CoV-2-N were harvested at the fifth and tenth generation of passage and identified by PCR. As shown in Figure 4, the presence of the SARS-CoV-2-N gene in the recombinant plasmid was confirmed by PCR, where a 1,702 bp band was detected. The presence of the SARS-CoV-2-S gene in the recombinant plasmid was confirmed by PCR, where a 4,306 bp band was detected. The PCR results show that the obtained genes were consistent with the expected fragment size. Based on these results, the recombinant viruses had been successfully rescued. Their exogenous protein expression was identified by Western blot. As shown in Figures 5A,B, the presence of SARS-CoV-2-S (141.2 kDa) and SARS-CoV-2-N (45.6 kDa) proteins in the recombinant virus was confirmed by Western blot analysis. In contrast, neither SARS-CoV-2-S nor SARS-CoV-2-N was detected in the mock and rPRV-Bartha-K61 group. In addition, the inserted genes SARS-CoV-2-S and SARS-CoV-2-N in the recombinant viruses were verified by sequencing. The sequencing result shows no mutation in SARS-CoV-2-S and SARS-CoV-2-N of the recombinant viruses at the fifth generation, proving that the recombinant viruses have good genetic stability.

### Growth Kinetics Analysis

**Figure 6:**
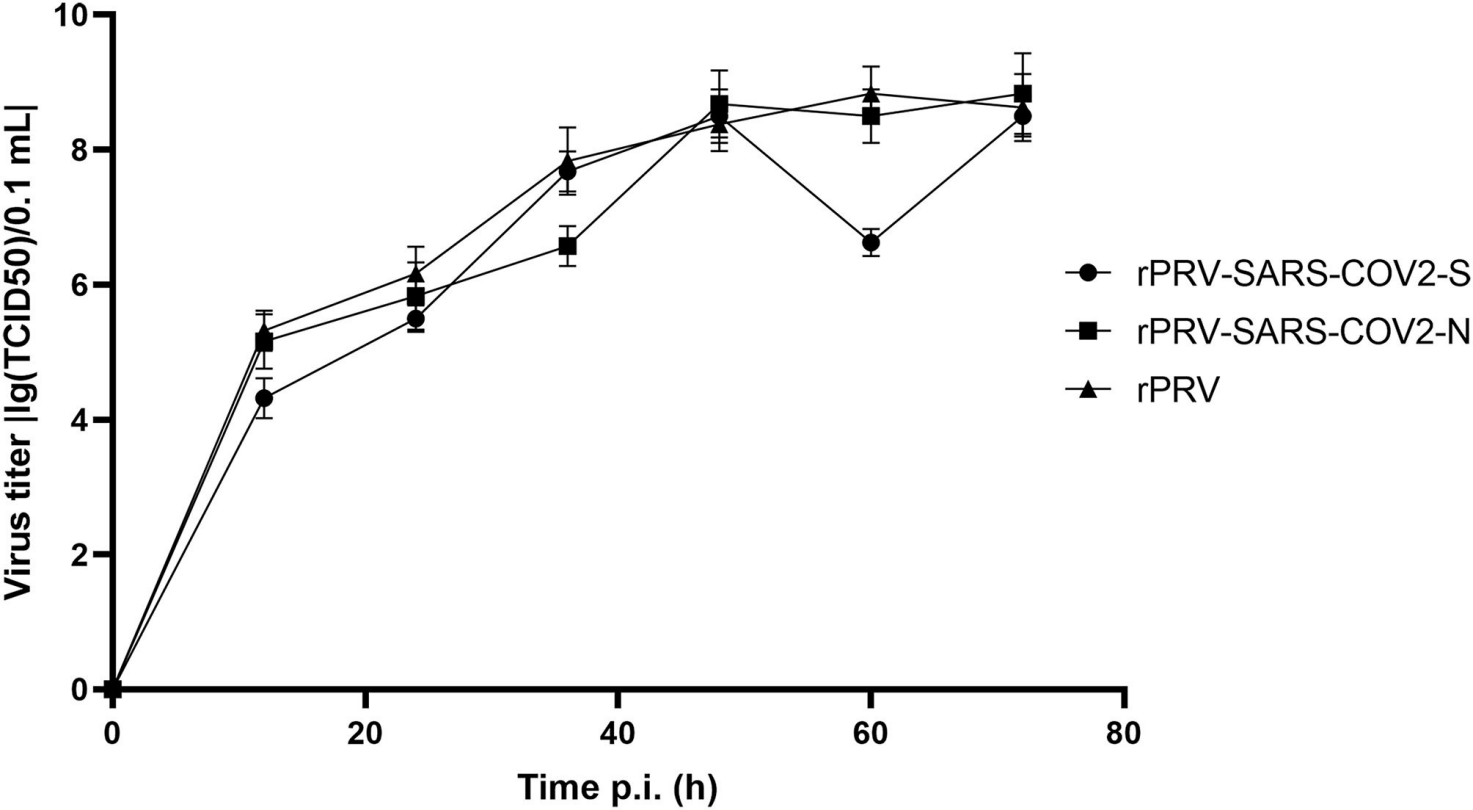
Growth kinetics of the recombinant virus and rPRV in PK-15 cells. Infected cells and supernatants were collected, and viral titers were determined at the indicated time points post-inoculation.

The TCID_50_ of PK-15 cells was determined at 12, 24, 36, 48, 60, and 72 h after inoculation. The results demonstrated that the recombinant virus rPRV-SARS-CoV-2-S and rPRV-SARS-CoV-2-N had a rapid rate of proliferation on the PK-15 cells. The peak value of the recombinant virus rPRV, rPRV-SARS-CoV-2-S, and rPRV-SARS-CoV-2-N titers was 1 ×10^8.2^ TCID_50_/0.1 mL, 1 ×10^8.4^ TCID_50_/0.1 mL and 1 ×10^8.9^ TCID_50_/0.1 mL, respectively. Though the recombinant virus rPRV-SARS-CoV-2-S has lower TCID50 than the other two groups at 60 h, its proliferation trend was similar to another group. The recombinant virus rPRV-SARS-CoV-2-N had the same proliferation characteristic as the rPRV, suggesting that the SARS-CoV-2-S and SARS-CoV-2-N gene insertion did not affect the virus replication *in-vitro* (Figure 6).

### Expression and Purification of SARS-CoV-2-N Protein

**Figure 7:**
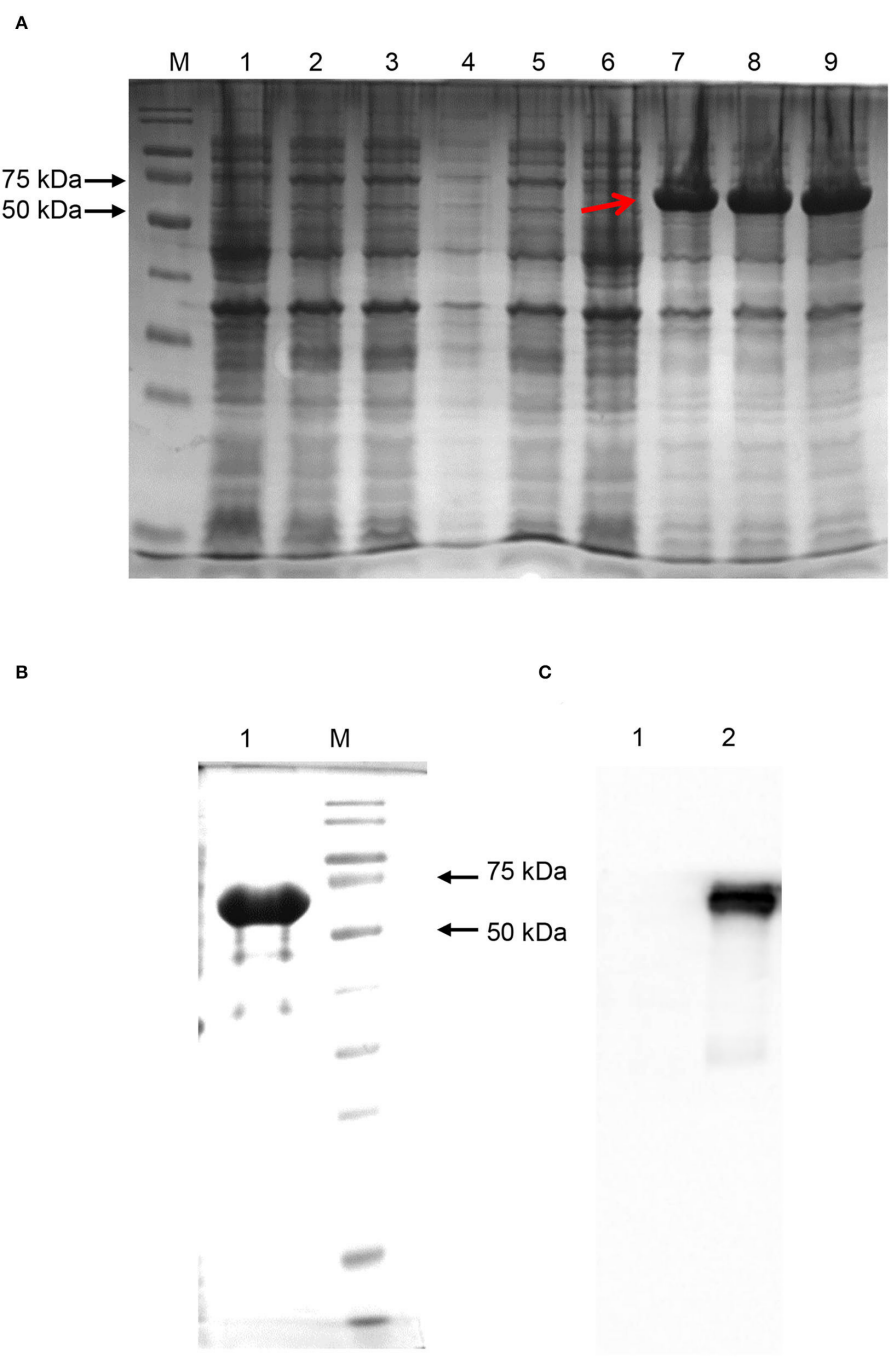
SARS-CoV-2-N expression and purification. **(A)** SARS-CoV-2-N expression. Proteins in Lanes 1, 2, and 3 were induced under the condition of IPTG concentration of 0.1 mM. Proteins in Lanes 4, 5, and 6 were induced under an IPTG concentration of 0.5 mM. Proteins in Lanes 7, 8, and 9 were induced under an IPTG concentration of 1 mM. The induction temperature of all the lanes was 20°C and the induction time was 16 h. The protein indicated by the red arrow had the best expression. **(B)** SARS-CoV-2-N purification. The protein was purified by Ni-NTA chromatography and eluted by Elution Buffer. **(C)** Western blotting by anti-SARS-CoV-2 N protein mice monoclonal antibodies (Sinobiological, Beijing).

When the IPTG concentration was 1 mM, the temperature was 20°C, and the induction time was 16 h, SARS-CoV-2-N was induced to the best expression (Figure 7A). As shown in Figures 7B,C, the N protein was successfully purified and identified by Western Blotting. The purified N protein was then used to develop I-ELISA assay.

### Development of SARS-CoV-2-S-Based and SARS-CoV-2-N-Based Indirect ELISA

Determined by CBT, the optimal working concentration of recombinant antigen (S protein) was 0.125 μg/Ml, while the optimal working concentration of antibody was 1:100. The optimal working concentration of antigen (N protein) was 0.5 μg/mL, while the working concentration of antibody was 1:1,000 (Tables 1, 2).

**Table 1:** The optimal working concentration of recombinant antigen (S protein) and antibody.

**Antigen concentration** **(ug/mL)**	**Antibody concentration**					
	**1:100**	**1:500**	**1:1,000**	**1:2,000**	**1:4,000**	**1:8,000**
2	3.80	3.33	2.08	1.35	0.81	0.77
1	5.70	4.95	2.84	1.47	0.66	0.58
0.5	7.23	5.72	2.72	1.15	0.57	0.54
0.25	8.17	6.10	2.61	1.07	0.54	0.49
0.125	**8.35**	6.37	3.07	1.32	0.60	0.28
0.0625	7.89	5.98	3.11	1.60	0.66	0.32

*The optimal working concentration of S protein was 0.125 μg/mL, while the optimal working concentration of the antibody was 1:100. The values indicated positive and negative (P/N) values in optical absorbance at 450 nm. The bold value is the maximum P/N value, and the corresponding concentration is the optimal working concentration*.

**Table 2:** The optimal working concentration of recombinant antigen (N protein) and antibody.

**Antigen concentration** **(ug/mL)**	**Antibody concentration**			
	**1:1,000**	**1:2,000**	**1:4,000**	**1:8,000**
8	2.86	2.06	1.00	0.53
4	4.96	3.10	1.40	0.68
2	6.26	3.99	1.36	0.67
1	6.35	3.97	1.57	0.65
0.5	**6.93**	3.98	1.61	0.68
0.25	6.65	4.13	1.71	0.74
0.125	6.54	3.5	2.39	0.97
0.0625	5.60	3.37	2.05	0.96

*The optimal working concentration of N protein was 0.5 μg/mL, the optimal working concentration of the antibody was 1:1,000. The values indicated positive and negative values (P/N) in optical absorbance at 450 nm. The bold value is the maximum P/N value, and the corresponding concentration is the optimal working concentration*.

### Antibody Detection of I-ELISA

After injection, the safety evaluation tests were conducted on mice. During the 28-day observation, mice in control, rPRV-SARS-CoV-2-S and rPRV-SARS-CoV-2-N groups were healthy without any clinical symptoms. However, mice in the rPRV group showed clinical symptoms, such as ataxia, on the fourth day, indicating the pathogenicity of PRV Bartha-K61 to mice was reduced, and the virulence was weakened because of the deletion of the TK gene.

**Figure 8:**
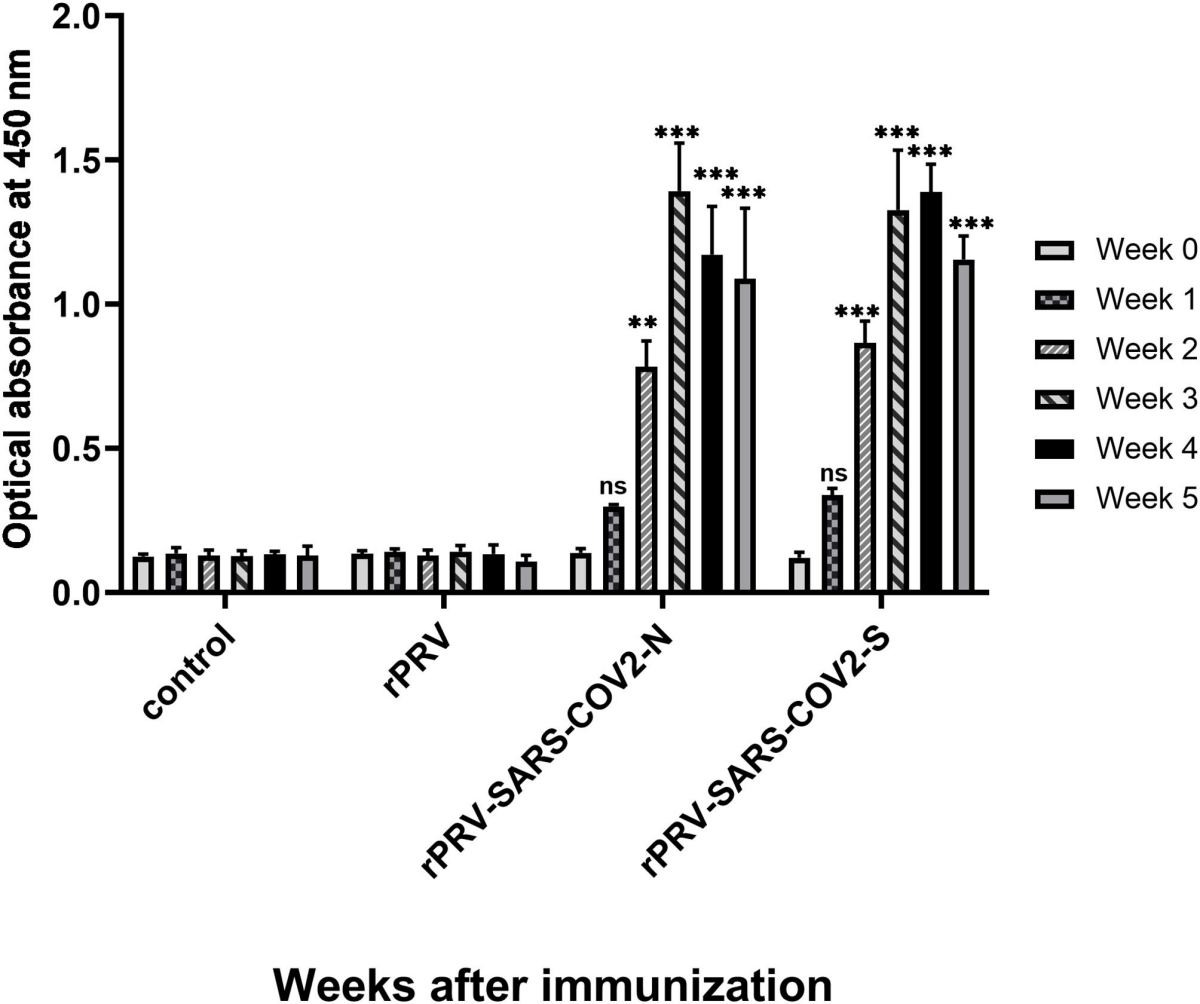
Total IgG level of mice immunized detection by I-ELISA. Compared with the control group (PBS group), total IgG levels in groups rPRV-SARS-CoV-2-S and rPRV-SARS-CoV-2-N increased significantly (*P* < 0.001) after week 2, but there was no significant change in rPRV group. ** and *** represented *P* value ≤ 0.01 and *P* value ≤ 0.001, respectively.

Total IgG level was detected in mice immunized with rPRV, rPRV-SARS-CoV-2-S, rPRV-SARS-CoV-2-N, and PBS by I-ELISA. The total IgG level of the mice immunized with rPRV-SARS-CoV-2-S and rPRV-SARS-CoV-2-N began to increase in the first week and was positive 2 weeks after the first immunization. Mice strengthened by the second immunization in week 2, and then the total IgG level of rPRV-SARS-CoV-2-N peaked at week 3, while the total IgG level of rPRV-SARS-CoV-2-S peaked at week 4. However, the total IgG level in the other groups showed no significant change from the first week (Figure 8).

## Discussion

Coronavirus is an important pathogen of humans and animals, which can infect humans or animals through the respiratory mucosal route. SARS-CoV-2 is the first global coronavirus in human history, which has brought high related medical costs to low-income and middle-income countries and huge losses to the world (25). The number of confirmed cases and deaths of SARS-CoV-2 continues to increase, and the virus continues to evolve and mutate, gradually forming a variety of mutant strains with increased transmissibility and pathogenicity, such as Delta strain and Omicron strain (26, 27). A study concluded that infection with the Delta variant had been detected in 96 countries worldwide (28). In addition, a study showed that the Omicron strain is more infectious and transmissible than the former strains because it contains many mutations (29). The immune-escaping strain has spread widely, making the epidemic more severe and SARS-CoV-2 a common health threat to humans.

SARS-CoV-2 will infect not only humans but also domestic animals, pets, and wild animals. Di Teodoro et al. have proved that respiratory tissues of cattle and sheep could be infected with SARS-CoV-2 by *ex-vivo* organ cultures (EVOC) experiments (30). Screening for seropositivity in pets of SARS-CoV-2-positive households in Italy showed that 3.3% of dogs and 5.8% of cats were seropositive (31). Zoos in the United States have reported cases of SARS-CoV-2 infection in wild animals, which are associated with infected zoo workers (32, 33). Therefore, SARS-CoV-2 has the potential to infect domestic animals, pets, and wild animals, and animals with or without clinical signs may reinfect humans through reverse zoonotic infection (34). It is essential to develop a vaccine that can induce the mucosal immune response and activate the humoral immune response, and the safe and effective recombinant virus vector vaccine can be the first choice.

S and N proteins are the most important protective antigens of the SARS-CoV-2 and SARS-CoV. SARS-CoV-2 has a similar S-RBD protein to SARS-CoV, which perfectly binds ACE2 (35). Herrera et al. found that S protein could produce neutralizing antibodies with strong immunogenicity and effectively prevent the replication of the virus *in-vivo* in infected mouse models (36). Mubarak et al. found that animals injected with the vaccine of the S protein RBD obtained better immune protection in animal experiments based on MERS-CoV (37). N protein, an internal protein of the virus, is the most conserved among the four structural proteins of SARS-CoV and is the most abundant in expression during infection (38). In addition, it can package the viral genome into helical ribonucleoprotein complexes and mediate viral RNA synthesis through RNA-binding protein domains, making it a candidate antigen in SARS-CoV vaccine studies (39, 40). Cai et al. performed arginine methylation of SARS-CoV-2-N protein and found that the protein could regulate RNA binding and suppress stress granule formation and viral replication (41). Zhao et al. found that N protein is not only an important B cell immunogen but also can induce a wide range of cellular immune responses (42).

PRV live vector vaccine can induce specific mucosal immunity, and PRV also has a wide host range, so the PRV Bartha-K61 strain was selected as the viral vector of recombinant virus vaccine in this study. The strain has been used worldwide for nearly 60 years for its safety, and it has the potential to become a mammalian vaccine carrier (18). In the genome, gE, gI, and TK genes are virulence-related genes but not necessary for replication (43, 44). Knockout of these genes can reduce the virulence of the virus, and these sites can serve as insertion sites for heterologous genes. The BAC-PRV Bartha-K61 infectious cloning operation platform was constructed in the previous work, and we replaced the TK gene with SARS-CoV-2-S and SARS-CoV-2-N genes, respectively, through this platform.

In this study, rPRV-SARS-CoV-2-S and rPRV-SARS-CoV-2-N recombinant viruses were successfully constructed by Red/ET recombinant technology. We also successfully rescue the recombinant viruses rPRV-SARS-CoV-2-S and rPRV-SARS-CoV-2-N on Vero cells. To ensure that the recombinant virus has stable proliferation in PK-15 cells, we compared the growth curve of recombinant viruses and the parent virus PRV-Bartha-K61. The proliferation trend of recombinant viruses was similar to the parent virus PRV-Bartha-K61 with minimal differences, indicating that the insertion of the foreign gene did not affect virus replication *in-vitro*.

The result of the Western blot showed that exogenous protein could be expressed. In addition, we successfully developed an I-ELISA based on the S and N proteins of SARS-CoV-2. Our data clearly showed that mice immunized with rPRV-SARS-CoV-2-S and rPRV-SARS-CoV-2-N produced a higher level of total IgG than mice injected with PBS and rPRV, indicating that recombinant viruses could induce total IgG antibodies. In general, these results suggested that the recombinant viruses rPRV-SARS-CoV-2-S and rPRV-SARS-CoV-2-N could express the S and N protein of SARS-CoV-2.

In conclusion, we developed a recombinant PRV expressing the SARS-CoV-2-S and SARS-CoV-2-N. This study can provide a reference for future research on live vector vaccines for domestic animals, pets, and wild animals and provide a method and strategy for the rapid preparation of an emergency live vaccine for SARS-CoV-2 and newly mutated viruses infectious diseases in the future.

## Data Availability Statement

The datasets presented in this study can be found in online repositories. The names of the repository/repositories and accession number(s) can be found in the article/Supplementary Material.

## Ethics Statement

The animal study was reviewed and approved by South China Agricultural University.

## Author Contributions

RL conducted the experiments, analyzed the data, and wrote the manuscript. GS conducted the experiments. JH helped with the animal experiment. ZX analyzed part of the data. XZ checked and finalized the manuscript. KF designed the experiments, modified the manuscript, and supervised the whole work. All authors read and approved the final manuscript.

## Funding

This study was supported by the Key Research and Development Program of Guangdong Province (2020B020222001), the Construction of Modern Agricultural Science and Technology Innovation Alliance in Guangdong Province (2020KJ128), the Guangdong Basic and Applied Basic Research Foundation (2019A1515012006), the National Natural Science Foundation of China (31902252), and the Special Project of National Modern Agricultural Industrial Technology System (CARS-41).

## Conflict of Interest

The authors declare that the research was conducted in the absence of any commercial or financial relationships that could be construed as a potential conflict of interest.

## Publisher's Note

All claims expressed in this article are solely those of the authors and do not necessarily represent those of their affiliated organizations, or those of the publisher, the editors and the reviewers. Any product that may be evaluated in this article, or claim that may be made by its manufacturer, is not guaranteed or endorsed by the publisher.
